# A pilot window-of-opportunity study of preoperative fluvastatin in localized prostate cancer

**DOI:** 10.1038/s41391-020-0221-7

**Published:** 2020-03-13

**Authors:** Joseph Longo, Robert J. Hamilton, Mehdi Masoomian, Najia Khurram, Emily Branchard, Peter J. Mullen, Mohamad Elbaz, Karen Hersey, Dianne Chadwick, Sangeet Ghai, David W. Andrews, Eric X. Chen, Theodorus H. van der Kwast, Neil E. Fleshner, Linda Z. Penn

**Affiliations:** 1 Princess Margaret Cancer Centre, University Health Network, Toronto, ON Canada; 2 Department of Medical Biophysics, University of Toronto, Toronto, ON Canada; 3 Division of Urology, Department of Surgical Oncology, University Health Network & University of Toronto, Toronto, ON Canada; 4 Department of Pathology, Laboratory Medicine Program, University Health Network, Toronto, ON Canada; 5 Joint Department of Medical Imaging, Mount Sinai Hospital & University Health Network, Toronto, ON Canada; 6 Sunnybrook Research Institute, Toronto, ON Canada

**Keywords:** Cancer therapy, Prostate cancer

## Abstract

**Background:**

Statins inhibit HMG-CoA reductase, the rate-limiting enzyme of the mevalonate pathway. Epidemiological and pre-clinical evidence support an association between statin use and delayed prostate cancer (PCa) progression. Here, we evaluated the effects of neoadjuvant fluvastatin treatment on markers of cell proliferation and apoptosis in men with localized PCa.

**Methods:**

Thirty-three men were treated daily with 80 mg fluvastatin for 4–12 weeks in a single-arm window-of-opportunity study between diagnosis of localized PCa and radical prostatectomy (RP) (ClinicalTrials.gov: NCT01992042). Percent Ki67 and cleaved Caspase-3 (CC3)-positive cells in tumor tissues were evaluated in 23 patients by immunohistochemistry before and after treatment. Serum and intraprostatic fluvastatin concentrations were quantified by liquid chromatography-mass spectrometry.

**Results:**

Baseline characteristics included a median prostate-specific antigen (PSA) level of 6.48 ng/mL (IQR: 4.21–10.33). The median duration of fluvastatin treatment was 49 days (range: 27–102). Median serum low-density lipoprotein levels decreased by 35% after treatment, indicating patient compliance. Median PSA decreased by 12%, but this was not statistically significant in our small cohort. The mean fluvastatin concentration measured in the serum was 0.2 μM (range: 0.0–1.1 μM), and in prostatic tissue was 8.5 nM (range: 0.0–77.0 nM). At these concentrations, fluvastatin induced PCa cell death in vitro in a dose- and time-dependent manner. In patients, fluvastatin treatment did not significantly alter intratumoral Ki67 positivity; however, a median 2.7-fold increase in CC3 positivity (95% CI: 1.9–5.0, *p* = 0.007) was observed in post-fluvastatin RP tissues compared with matched pre-treatment biopsy controls. In a subset analysis, this increase in CC3 was more pronounced in men on fluvastatin for >50 days.

**Conclusions:**

Fluvastatin prior to RP achieves measurable drug concentrations in prostatic tissue and is associated with promising effects on tumor cell apoptosis. These data warrant further investigation into the anti-neoplastic effects of statins in prostate tissue.

## Introduction

Statins are potent and specific inhibitors of HMG-CoA reductase (HMGCR), the rate-limiting enzyme of the mevalonate pathway [1]. For decades, statins have been prescribed for the chronic management of hypercholesterolemia; however, mounting epidemiological evidence supports a possible role for these drugs in the prevention of advanced prostate cancer (PCa) and improved patient outcomes [2–8].

Data from pre-clinical studies have demonstrated that statins can induce tumor-specific apoptosis by directly inhibiting HMGCR in a number of different cancer types, including PCa [9–11]. These anti-cancer effects have been attributed to both cholesterol-dependent and -independent mechanisms downstream of HMGCR inhibition [8, 11, 12].

Taken together, these data suggest that statins may have therapeutic utility in the neoadjuvant setting and potentially offer an immediate and inexpensive opportunity to reduce PCa morbidity and mortality. Here, we report the results of a pilot window-of-opportunity clinical trial aimed to evaluate the effects of neoadjuvant fluvastatin on markers of cell proliferation and apoptosis in men with localized PCa.

## Materials and methods

### Patients

We conducted an open-label, single-institution, phase II single-arm study of neoadjuvant fluvastatin in men with localized PCa (ClinicalTrials.gov: NCT01992042). All procedures were approved by the Research Ethics Board of Princess Margaret Cancer Centre and participants provided informed consent. Eligible men (recruited between March 2014 and June 2016) had surgically resectable, localized prostate adenocarcinoma, confirmed histologically by a transrectal ultrasound-guided biopsy performed within 12 months of enrollment; Gleason score 6 disease with high risk for pathological upgrading at radical prostatectomy (RP) (≥3 positive cores, prostate volume <40 cm^3^ and >30% involvement of one biopsy core) or Gleason score ≥7 (involving at least 30% of one unfragmented biopsy core); normal organ/marrow function; and Eastern Cooperative Oncology Group performance status of 0–1.

Exclusion criteria included current/previous neoadjuvant or hormonal management of PCa; use of 5-alpha reductase inhibitors within 6 months of study enrollment; statin use within 6 months of PCa diagnosis; history of pelvic radiation or bilateral orchiectomy, adrenalectomy or hypophysectomy; and previous history of malignancy other than PCa or treated squamous/basal cell carcinoma of the skin within 5 years of enrollment.

### Study design

Patients were prescribed 80 mg fluvastatin (40 mg twice per day, orally) for a period of 4–12 weeks, depending on time from consent to surgery. Patients’ diagnostic biopsy samples served as pre-intervention controls to examine the effects of fluvastatin treatment after the prostate was removed.

Blood and serum analyses were conducted pre- and post-fluvastatin treatment for all participants to determine the effects of fluvastatin on serum biochemistry (including cholesterol levels to assess compliance to treatment), prostate-specific antigen (PSA) levels and serum hormones. During the intervention period, patients were also monitored via telephone call to document any severe adverse reactions, new concomitant medication use and/or treatment compliance. Compliance was further determined from the number of tablets returned on the day of surgery and the total number of days on treatment.

### Sample procurement

Prostate needle biopsies taken within 12 months of enrollment were reviewed, and the most representative cores (same side as the largest tumor in corresponding RP and with the largest amount of tumor with Gleason ≥6) were selected by the study pathologist for analysis. Immediately after surgery, RP specimens were weighed, measured and inked according to standard University Health Network (UHN) protocol. Specimens were then sectioned and inspected for grossly identifiable tumor. Tissue samples from the identifiable tumor and normal prostate parenchyma were flash-frozen as per standard UHN tissue banking procedures, and additional tissue samples were formalin-fixed and processed for paraffin embedding. The remaining tissue was processed according to the College of American Pathologists’ guidelines for diagnostic purposes.

### Tissue microarrays

Tissue microarray (TMA) blocks were constructed by UHN pathologists according to standard protocols. First, hematoxylin and eosin slides of the biobanked paraffin blocks were reviewed by the study pathologist for construction of the TMA; however, in cases where the biobanked paraffin blocks did not contain sufficiently representative tumor, TMA cores were obtained from the diagnostic paraffin blocks. Triplicate 1 mm cores were obtained from donor paraffin blocks with the largest tumor foci, or from the largest foci in both the left and right prostatic lobes in cases with bilateral disease.

### Endpoint measurements

The primary endpoint under investigation was the effect of fluvastatin on the proliferation index of the tumor, as evaluated by immunohistochemistry (IHC) for the marker Ki67. The secondary endpoint was the effect of fluvastatin on tumor cell apoptosis, as evaluated by IHC for the marker cleaved Caspase-3 (CC3). Four micron sections from the biopsy cores and TMAs were stained using specific antibodies against Ki67 (Agilent Dako, M7240), CC3 (Cell Signaling Technology, #9661), or ERG (Biocare Medical, CM421A) by the Pathology Research Program laboratory, Department of Pathology, UHN. ERG expression was evaluated to verify that the matched biopsy and RP tissues being compared represented similar tumor foci. For Ki67 and CC3, triplicate fields of ~200 tumor cells each were scored for the percentage of positive cells. This method is similar to those previously used to evaluate Ki67 immunostaining in PCa tissues [13, 14]. Serum/intraprostatic fluvastatin concentrations were measured by high performance liquid chromatography (HPLC)-tandem mass spectrometry (MS/MS), as described previously [11]. For intraprostatic fluvastatin measurements, 30–200 mg of flash-frozen tissue was processed and analyzed.

### Statistical analysis

The primary aim of this study was to compare the tumor Ki67 proliferation index before and after fluvastatin treatment, with a decrease in Ki67 considered a response. A sample size of 40 was originally planned, which was based on 2 published window-of-opportunity trials of preoperative statin treatment in breast cancer [15, 16]. Due to slow accrual, 33 patients completed the study and 23 were evaluable for immunohistochemical outcomes. Pre- and post-fluvastatin statistical comparisons were made using Wilcoxon matched-pairs signed rank tests. A *p* value of less than 0.05 was considered statistically significant.

### Live-cell imaging

Details can be found in “Supplementary Information.”

## Results

**Fig. 1:**
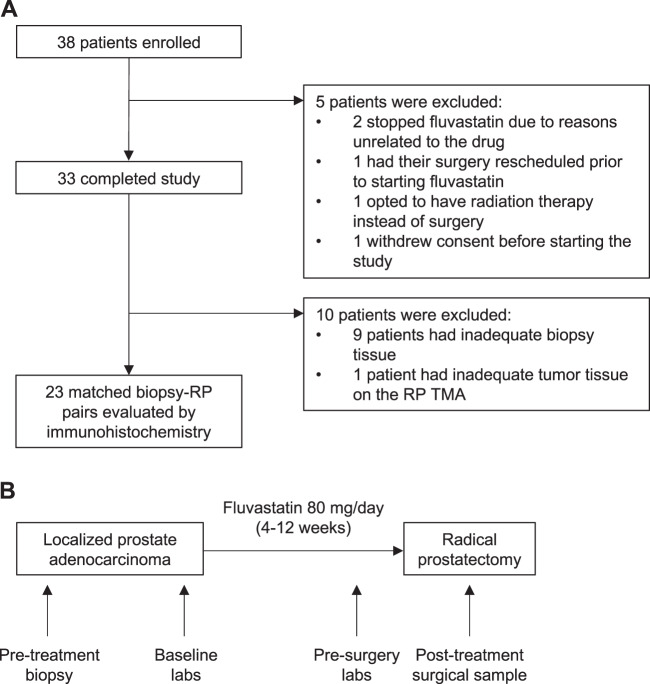
Study overview. **a** CONSORT diagram and **b** schematic of the study.

Thirty-eight patients diagnosed with localized prostate adenocarcinoma were enrolled, and 33 patients completed the study (Fig. 1). Five patients were excluded from the study after enrollment (Fig. 1). Baseline characteristics of the 33 patients who completed the study are summarized in Table 1.

**Table 1:** Baseline characteristics.

Baseline characteristics	
Median age, years (range)	62 (51–75)
Median BMI, kg/m^2^ (IQR)	27.2 (24.0–30.2)
Smoking, *n* (%)	
Never	14 (42.4%)
Previous	13 (39.4%)
Current	6 (18.2%)
Hypertension, *n* (%)	1 (3%)
Diabetes, *n* (%)	0 (0%)
Median PSA, ng/mL (IQR)	6.48 (4.21–10.33)
D’Amico risk classification, *n* (%)	
Low risk	2 (6%)
Intermediate risk	29 (88%)
High risk	2 (6%)
Biopsy Gleason score, *n* (%)	
6 (3 + 3)	2 (6%)
7 (3 + 4)	26 (79%)
7 (4 + 3)	5 (15%)
Clinical stage, *n* (%)	
T1	17 (52%)
T2 or greater	16 (48%)

**Fig. 2:**
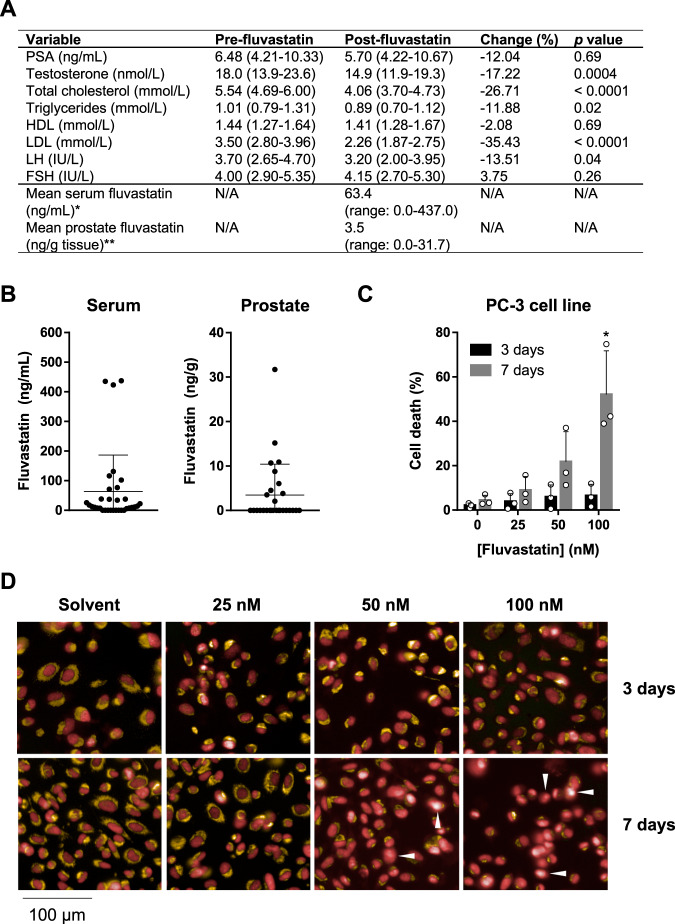
Serum and tissue measurements before and after fluvastatin treatment, and effects of fluvastatin on PCa cell death. **a** Median (IQR) values are reported, unless otherwise indicated. Statistical comparisons (*p* values) are the result of Wilcoxon matched-pairs signed rank tests. *LLOQ: 5 ng/mL; **LLOQ: 0.25 ng/g; 28 of the 33 enrolled patients were evaluated. HDL high-density lipoprotein, LH luteinizing hormone, FSH follicle-stimulating hormone, N/A not applicable. **b** Fluvastatin concentrations in the serum and prostate tissue of patients were quantified by HPLC-MS/MS. Data are represented as the mean ± SD. **c** PC-3 cells were treated with a range of fluvastatin concentrations measurable in prostatic tissue (0–100 nM) for 3 or 7 days, and cell death was quantified using live-cell imaging analysis. Data are represented as the mean + SD, *n* = 3. *p < 0.05 (one-way ANOVA with Bonferroni’s multiple comparisons test, where each group was compared with the corresponding solvent control). **d** Representative PC-3 live-cell images. Treated cells were stained with DRAQ5 (red) to identify all nuclei in the well and to score nuclear condensation in response to fluvastatin treatment, as well as TMRE (orange) to identify cells with healthy and active mitochondria. Nuclear condensation and the loss of TMRE staining are markers of cell death. Examples of dead cells are indicated by the white arrows.

The pathologic and clinical outcome data are summarized in Table 2. The median duration of fluvastatin treatment was 49 days (range: 27–102). Three patients continued fluvastatin treatment for longer than 12 weeks (90–102 days). For two of these patients, a surgery timeslot was not available within the 4–12 weeks treatment timeframe. The third patient postponed their surgery date for personal reasons. The median compliance to treatment was 99%. Compliance was estimated from the number of fluvastatin tablets returned on the day of surgery and the total number of days on treatment. Compliance was also assessed biochemically by measuring serum cholesterol levels (Fig. 2a). There were statistically significant decreases in both total cholesterol and low-density lipoprotein (LDL) levels after fluvastatin treatment (27% and 35%, respectively), confirming that the patients were compliant. Serum PSA and hormone levels at baseline and after fluvastatin treatment are summarized in Fig. 2a. Median PSA declined by 12%, but this decrease was not statistically significant in our small cohort. Significant decreases in luteinizing hormone (14%) and testosterone (17%) were noted, but levels of both hormones remained within their normal physiological range.

**Table 2:** Pathologic and clinical outcomes.

Variable	
Median fluvastatin duration, days (range)	49 (27–102)
Median compliance, % (range)	99% (78–100%)
Pathologic stage, *n* (%)	
pT0	0 (0%)
pT1	0 (0%)
pT2	16 (48%)
pT3	17 (52%)
pT4	0 (0%)
Extraprostatic extension, *n* (%)	15 (45%)
Positive margins, *n* (%)	9 (27%)
Positive nodes, *n* (%)	
pN0	20 (61%)
pN1	2 (6%)
pNX	11 (33%)
RP Gleason score, *n* (%)	
5 (2 + 3 or 3 + 2)	3 (9%)
6 (3 + 3)	4 (12%)
7 (3 + 4)	22 (67%)
7 (4 + 3)	3 (9%)
8 (4 + 4)	1 (3%)
Change from baseline Gleason score, *n* (%)	
Upgraded	1 (3%)
No change	26 (79%)
Downgraded	6 (18%)
Median follow-up, months (range)	36 (8–52)
BCR and/or use of RT and/or ADT, *n* (%)	
BCR^a^	6 (18%)
Salvage RT	6 (18%)
Adjuvant RT	2 (6%)
ADT	3 (9%)
Metastatic CRPC, *n* (%)	1 (3%)

*CRPC* castration-resistant PCa.

^a^BCR = two consecutive PSA values above 0.2 ng/mL, receipt of salvage radiation therapy (RT) and/or receipt of androgen deprivation therapy (ADT).

HPLC-MS/MS was performed to quantify the concentration of fluvastatin achieved in the serum and prostate after treatment (Fig. 2a, b). Due to limited tissue availability, intraprostatic fluvastatin concentrations were evaluated in 28 patients. Fluvastatin was measurable (above the lower limit of quantification (LLOQ)) in the serum of 24 patients (73%) and in the prostate of 10 patients (36%). The mean concentration of fluvastatin measured in the serum was 63.4 ng/mL or 0.2 μM (range: 0.0–437.0 ng/mL or 0.0–1.1 μM), and in prostatic tissue was 3.5 ng/g or ~0.0085 μM (range: 0.0–31.7 ng/g or 0.0–0.077 μM). When considering only the patients where fluvastatin was measurable, the mean fluvastatin concentration in the serum was 87.2 ng/mL or 0.21 μM, and in the prostate was 9.7 ng/g or ~0.024 μM.

To evaluate whether these physiologically achievable concentrations of fluvastatin were sufficient to induce PCa cell death in vitro, we treated PC-3 cells with 0–100 nM fluvastatin for 3 or 7 days, and then quantified cell death using live-cell imaging. We chose to evaluate PC-3 cells because we previously characterized this cell line as statin sensitive [11]. We observed a dose- and time-dependent increase in cell death (Fig. 2c, d), suggesting that physiologically achievable concentrations of fluvastatin can have anti-cancer effects in vitro given sufficient exposure time.

**Fig. 3:**
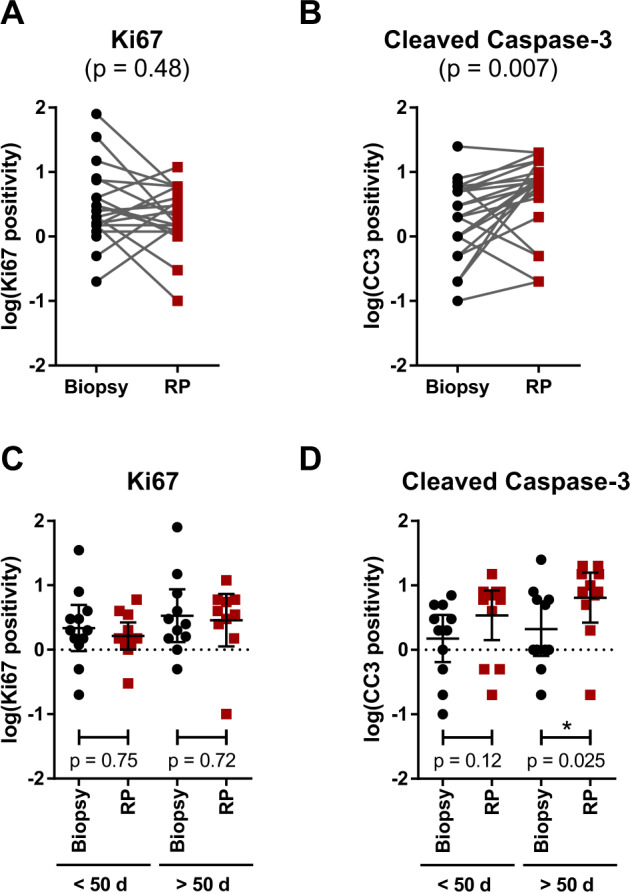
Effects of fluvastatin on intratumoral proliferation and apoptosis. Percentage of (**a**) Ki67- and (**b**) cleaved Caspase-3 (CC3)-positive tumor cells in pre-treatment biopsy and post-fluvastatin RP tissues (log transformed). Patients were subsequently subdivided based on the duration of fluvastatin treatment (<50 or >50 days). **c** Ki67 and **d** CC3 positivity in pre-treatment biopsy and post-fluvastatin RP tissues (log transformed), subdivided based on the duration of fluvastatin treatment. Data are represented as the mean ± 95% CI. Statistical comparisons (*p* values) are the result of Wilcoxon matched-pairs signed rank tests.

We next went on to evaluate whether fluvastatin treatment altered intratumoral markers of proliferation and/or apoptosis in our cohort of patients. Ten patients were excluded from these analyses due to inadequate tumor tissue in either the pre-treatment biopsy or post-treatment RP specimen, thus reducing our dataset to 23 patients. Given the multifocal nature of PCa, we first probed the biopsy and RP tissues for ERG expression to ensure the pre- and post-fluvastatin tissues being compared represented similar tumor foci. All 23 matched biopsy and RP samples were concordant for ERG expression (18 were ERG-negative and 5 were ERG-positive), providing confidence that similar tumor foci were being compared before and after treatment. We then proceeded to evaluate the endpoints of our study. No significant change in proliferation index was observed post-fluvastatin treatment, as determined by Ki67 staining (Fig. 3a); however, we observed highly variable Ki67 staining in the pre-treatment biopsies (Supplementary Fig. 1). A median Ki67 positivity of 2.0% (interquartile range (IQR): 1.5–4.0%) was observed in the pre-treatment samples. Four patients had higher than 4.0% Ki67-positive cells at baseline (range: 7.5–80.0%); however, these patients did not seem to have more aggressive disease in terms of Gleason score, clinical stage, or biochemical recurrence (BCR), and three of the four patients were negative for ERG expression. A median Ki67 positivity of 2.0% (IQR: 1.2–4.0%) was similarly observed in post-treatment RP tissues, and the median fold change in Ki67 positivity was 0.8 (95% confidence interval (CI): 0.3–2.0, *p* = 0.48).

We observed a median 2.7-fold increase in CC3 staining post-treatment (95% CI: 1.9–5.0, *p* = 0.007) (Fig. 3b), where a trend toward a greater increase was observed in patients with a greater decline in serum LDL (Spearman *r* = −0.33; 95% CI: −0.67–0.12, *p* = 0.13) (Supplementary Fig. 2). The median CC3 positivity in biopsy and RP tissues was 2.0% (IQR: 1.0–5.0%) and 7.0% (IQR: 4.0–10.0%), respectively. One patient had high basal CC3 staining (25% CC3-positive cells) that remained high (20% CC3-positive cells) after treatment (Supplementary Fig. 1); however, all other pathologic and clinical features were unremarkable.

To provide confidence that this increase in apoptosis was due to fluvastatin treatment, and not due to other factors such as tissue heterogeneity or damage caused by the biopsy/RP procedures, we stained independent TMAs comprised of RP tissues from 24 statin-naïve, intermediate-risk patients treated at UHN, with similar pathologic/clinical characteristics as the patients in our study (Supplementary Table 1). In these tissues, we observed a median CC3 positivity of 0.5% (IQR: 0.2–1.9%), which was comparable with the CC3 staining in our pre-treatment biopsies and less than what we observed after fluvastatin treatment.

In a subset analysis, we divided the patients around the median fluvastatin duration (<50 days vs. >50 days) (Fig. 3c, d). Intratumoral Ki67 positivity remained unchanged, even in patients on treatment for >50 days (median 0.8-fold change (95% CI: 0.3–4.0) in men on fluvastatin for <50 days (*p* = 0.75), and median 0.2-fold change (95% CI: 0.03–2.5) in men on fluvastatin for >50 days (*p* = 0.72)) (Fig. 3c). For CC3, we observed a median 2.3-fold increase (95% CI: 1.4–8.0, *p* = 0.12) after treatment in men on fluvastatin for <50 days, whereas a median 3.3-fold increase (95% CI: 0.8–8.0, *p* = 0.025) was observed in men on fluvastatin for >50 days (Fig. 3d).

## Discussion

The goal of this study was to determine whether neoadjuvant fluvastatin treatment had anti-proliferative and/or pro-apoptotic effects in PCa patients. Here, we provide evidence that short-term (4–12 weeks) fluvastatin treatment at a cholesterol-lowering dose prior to RP can increase the percentage of apoptotic PCa cells in the tumor relative to baseline. While pre-clinical studies have demonstrated that statins can induce PCa apoptosis in vitro and in animal models [10, 11, 17], this is the first prospective study to support that this same effect can be achieved in PCa patients at a typical cholesterol-lowering dose.

While this paper was in preparation, a complementary study evaluated the effects of preoperative atorvastatin treatment versus placebo in men with localized PCa [18]. In this study by Murtola et al., atorvastatin treatment did not significantly alter tumor proliferation index overall, but the researchers observed a 14% reduction in Ki67 staining in men on atorvastatin for at least 28 days [18]. In our study, fluvastatin treatment did not significantly affect intratumoral Ki67 positivity, even when accounting for treatment duration (Fig. 3a, c); however, highly variable Ki67 staining in our pre-treatment biopsies coupled with our low sample size made it difficult to reliably score overall changes in Ki67 positivity (Supplementary Fig. 1).

Consistent with previous observational data [19], we measured a 12% decrease in serum PSA levels after fluvastatin treatment (Fig. 2a), but this decrease was not statistically significant in our small cohort of patients. In the trial by Murtola et al., a decrease in serum PSA in response to atorvastatin was observed in high-grade patients [18]; however, our limited sample size precluded a similar subgroup analysis.

Interestingly, while we observed that six patients were downgraded in Gleason score at RP compared with biopsy, only one patient was upgraded to Gleason 8 (Table 2). The reported incidence of upgrading from Gleason 6 or less to ≥7 is ~35%, and is usually more common than downgrading [20, 21]. Whether this observation was due to fluvastatin treatment or merely due to small sample size bias remains to be determined; however, epidemiological studies have reported that statin users have a reduced advanced PCa risk [2, 5]. Although this trial was not designed nor powered to evaluate the effects of fluvastatin treatment on BCR, six patients in our study relapsed within 36 months following RP (Table 2). Tissues from five of these patients were evaluated by IHC. Interestingly, these patients were not among those that had high basal Ki67, and four of the five patients had at least a threefold increase in CC3 positivity after treatment. Larger, placebo-controlled trials are needed to evaluate the long-term effects of preoperative fluvastatin treatment on pathologic and clinical outcomes.

We demonstrated for the first time that fluvastatin can be measured in the prostate after 4–12 weeks of fluvastatin treatment. The concentrations we report here are comparable with those recently reported for atorvastatin [22]. Similar to the atorvastatin study, we were unable to measure fluvastatin in the prostate of every patient. Intraprostatic fluvastatin concentrations were below the LLOQ in 18 patients (64%), and yet serum fluvastatin was measurable in 12 of these patients. This could be due to variable times between last fluvastatin dose, blood draw, and RP, which were not standardized. This is particularly important because, unlike atorvastatin [22], fluvastatin did not accumulate in the prostate relative to the serum (Fig. 2a, b). Future studies are required to evaluate the achievable concentrations of different statins in the prostate, which may help to inform which statin offers the greatest anti-PCa potential.

Importantly, daily fluvastatin treatment allowed for the drug to reach the prostate at nanomolar concentrations. Fluvastatin concentrations at the higher end of the achievable range induced PCa cell death in vitro in a time-dependent manner (Fig. 2c, d), which is consistent with statin-induced apoptosis being both dose- and time-dependent [11, 23–25]. In line with these data, we observed increased tumor cell apoptosis following 4–12 weeks of fluvastatin treatment in our cohort of patients, with a greater increase observed in patients on fluvastatin for >50 days (Fig. 3b, d). These data suggest that longer exposure to concentrations of fluvastatin achievable in the prostate may be equally as effective at inducing apoptosis as shorter exposure to higher concentrations. We also observed a trend toward increased apoptosis in patients with a greater decline in serum LDL (Supplementary Fig. 2), but this was not statistically significant in our small cohort and requires further validation.

Collectively, the results of this pilot study reveal that neoadjuvant fluvastatin treatment prior to RP may be effective at inducing intratumoral apoptosis in men with localized PCa. These results may underlie retrospective evidence suggesting benefit from statins in reducing PCa progression and mortality. Larger, placebo-controlled trials are required to validate these results and to evaluate the potential long-term benefit of neoadjuvant statin therapy in PCa.

## Supplementary information

Supplementary Information
