# Supplementary material for: A pilot window-of-opportunity study of preoperative fluvastatin in localized prostate cancer
Source: Prostate Cancer Prostatic Dis. 2020 Mar 13;23(4):630–7. doi: 10.1038/s41391-020-0221-7 (PMC7655503; doi:10.1038/s41391-020-0221-7)
Supplement: Supplementary file 1 — Supplementary Information [file 41391_2020_221_MOESM1_ESM.pdf]

SUPPLEMENTARY INFORMATION

MATERIALS & METHODS

PC-3 live-cell imaging and analysis

PC-3 cells were purchased from the American Type Culture Collection (ATCC) and maintained in RPMI 1640 supplemented with 10% fetal bovine serum (FBS), 100 units/mL penicillin and 100 μg/mL streptomycin. Cells were routinely confirmed to be mycoplasma-free using the MycoAlert Mycoplasma Detection Kit (Lonza), and their authenticity was verified by short tandem repeat (STR) profiling. Cells were seeded at 150 cells/well (7-day assay) or 875 cells/well (3-day assay) in a CellCarrier-384 Ultra Microplate (PerkinElmer). Approximately 24 hours later, cells were treated with the indicated concentrations of fluvastatin (US Biological) or ethanol as a solvent control. 30 minutes prior to imaging, the cells were stained with DRAQ5 (1 μM; BioStatus) and tetramethylrhodamine ethyl ester perchlorate (TMRE) (2 μM; Life Technologies). Cells were then imaged using an Opera Phenix automated confocal microscope (PerkinElmer) with a 20x air objective. Nine different fields of view for each well were acquired. Two or three technical replicates per treatment condition were performed for each of three independent experiments. Image acquisition, calculation of intensity features for each channel and image analysis were performed using the Harmony high-content imaging and analysis software v4.9 (PerkinElmer). DRAQ5 staining was used to identify all nuclei in the well and to score nuclear condensation in response to treatment, whereas TMRE was used to identify cells with healthy and active mitochondria. The image data sets were then subjected to linear classification analysis using PhenoLOGIC™ to determine the percentage of dead cells per treatment condition. Briefly, training sets consisting of approximately 20 cells per population ("viable" or "dead") were generated, and phenotypic parameters and intensity features were calculated for every cell. The software then calculated a multi-parametric linear classifier optimized to discriminate between "viable" and "dead" cells, and the percentage of cells classified as "dead" was calculated per treatment condition.

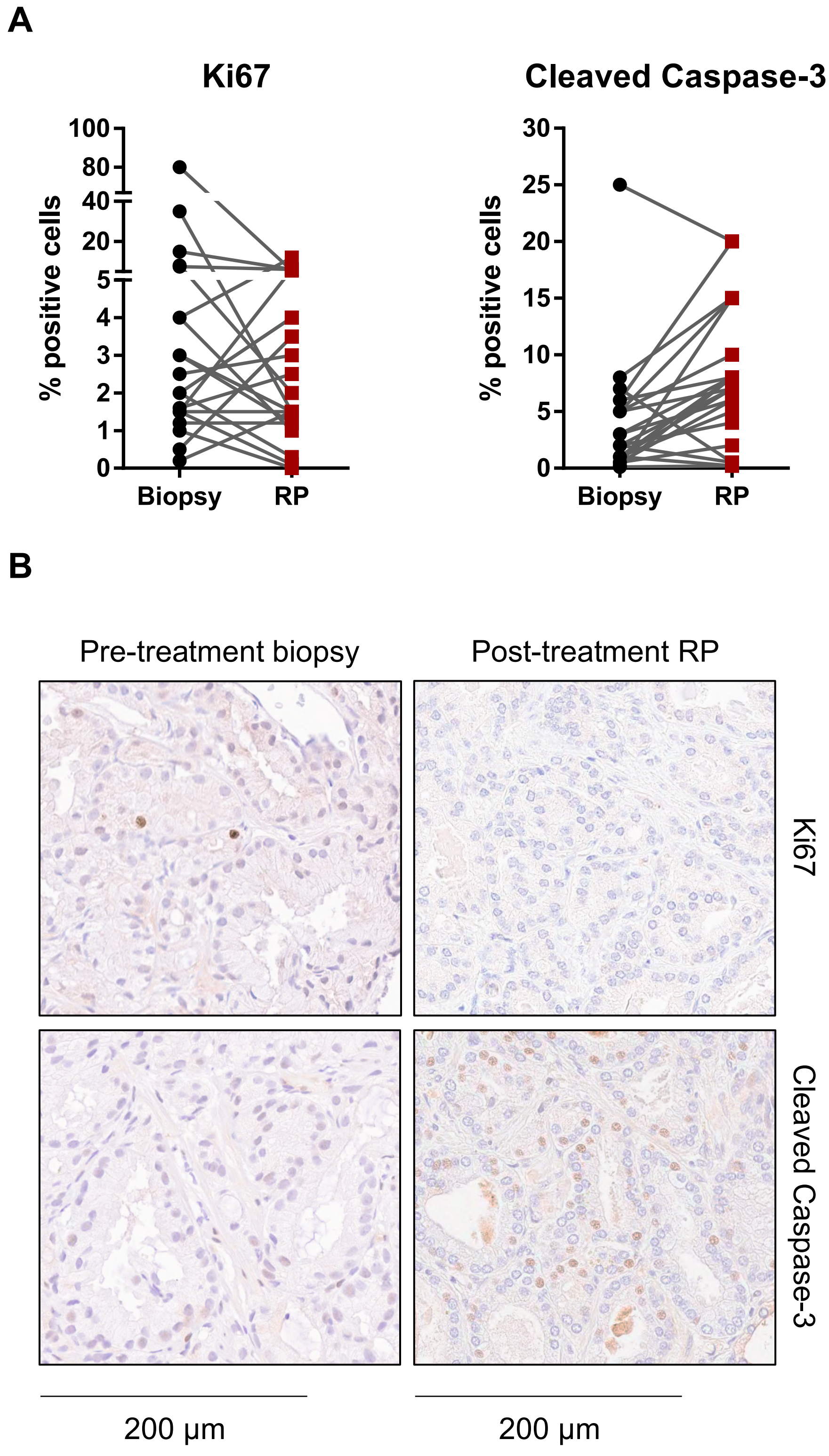

Supplementary Figure 1: (**A**) Percentage of Ki67- and cleaved Caspase-3 (CC3)-positive tumor cells in pre-treatment biopsy and post-fluvastatin RP tissues. (**B**) Representative images of Ki67 and CC3 staining in a matched biopsy and RP tissue pair.

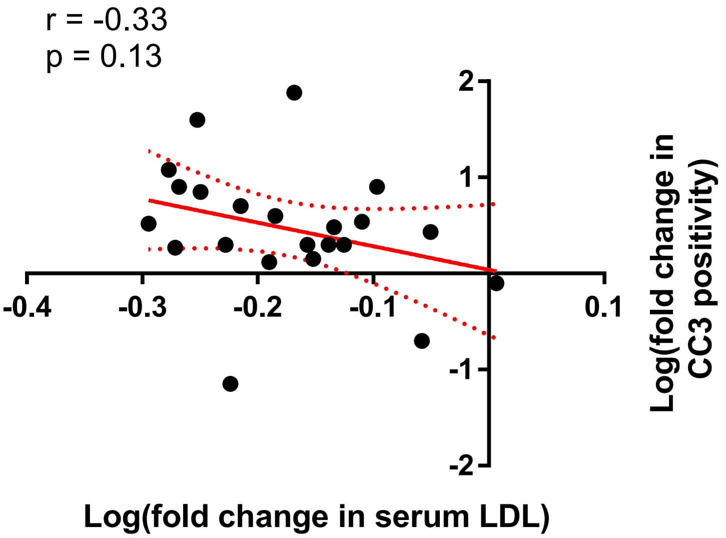

Supplementary Figure 2: Correlation between the fold change in intratumoral cleaved Caspase-3 (CC3) positivity and the fold change in serum LDL levels in response to fluvastatin treatment (Spearman r = -0.33; 95% CI: -0.67-0.12, p = 0.13). Line of best fit with 95% CI is shown in red.

Supplementary Table 1: Baseline characteristics & pathologic and clinical outcomes of statin-naïve control cohort.

Baseline characteristics	
Median age, years (range)	75 (61-87)
Median PSA, ng/mL (IQR)	7.80 (5.12-11.72)
D'Amico risk classification, *n* (%)	
Low risk	0 (0%)
Intermediate risk	24 (100%)
High risk	0 (0%)
Biopsy Gleason score, *n* (%)	
6 (3 + 3)	1 (4%)
7 (3 + 4)	14 (58%)
7 (4 + 3)	9 (38%)
Clinical stage, *n* (%)	
T1	10 (42%)
T2 or greater	14 (58%)
Pathologic and clinical outcomes	
Pathologic stage, *n* (%)	
pT0	0 (0%)
pT1	0 (0%)
pT2	11 (46%)
pT3	13 (54%)
pT4	0 (0%)
Extraprostatic extension, *n* (%)	14 (58%)
Positive margins, *n* (%)	3 (13%)
Positive nodes, *n* (%)	
pN0	11 (46%)
pN1	0 (0%)
pNX	13 (54%)
RP Gleason score, *n* (%)	
5 (2 + 3 or 3 + 2)	0 (0%)
6 (3 + 3)	1 (4%)
7 (3 + 4)	9 (38%)
7 (4 + 3)	12 (50%)
8 (4 + 4)	1 (4%)
9 (4 + 5)	1 (4%)
Change from baseline Gleason score, *n* (%)	
Upgraded	2 (8%)
No change	22 (92%)
Downgraded	0 (0%)
